# Formulation and characterization of starch-based novel biodegradable edible films for food packaging

**DOI:** 10.1007/s13197-023-05803-2

**Published:** 2023-08-19

**Authors:** Chetana Shanbhag, Ramnath Shenoy, Prakasha Shetty, M. Srinivasulu, Ramakrishna Nayak

**Affiliations:** 1 Department of Chemistry, Manipal Institute of Technology, Manipal Academy of Higher Education, Manipal, Karnataka 576104 India; 2 Department of Humanities and Management, Manipal Institute of Technology, Manipal Academy of Higher Education, Manipal, Karnataka 576104 India; 3 Department of Chemistry, Alva’s Institute of Engineering and Technology, Moodbidri, Karnataka 574225 India

**Keywords:** Edible films, Food packaging films, Starch-based films, Biodegradable films

## Abstract

Petroleum-based plastics were widely used as packaging materials. However, plastic materials were not reusable and biodegradable, causing a severe negative impact on the environment. Edible films can be a suitable alternative to plastic films, particularly in food packaging. This research work prepared edible films containing blends of cornstarch, arrowroot powder, refined wheat flour, vinegar, and glycerol. Arrowroot powder added strength and nutritional value to the films. Glycerol, as a plasticiser, improved the flexibility of films. The combination of vinegar and glycerol increased the film’s strength. The characteristic properties of prepared films, like thickness, bursting strength, moisture content, transparency, water-solubility, water vapour permeability, tensile strength, elongation, and Young’s modulus, were analysed. The thermal stability of the films was evaluated by thermogravimetric analysis. The films were characterised by FTIR spectroscopy, and their surface morphology was analysed by scanning electron microscopy. The prepared films exhibited excellent properties suitable for food packaging.

**Supplementary Information:**

The online version contains supplementary material available at 10.1007/s13197-023-05803-2.

## Introduction

The primary concern of food packaging technology is to retain the food quality over a crucial period while addressing other challenges such as material costs and energy, increased environmental awareness, and solid waste disposal laws. One of the latest methods to enhance food quality is the usage of edible films. Because of their benefits over synthetic films, edible films have attracted much interest. One of the essential benefits of edible packaging over synthetic packaging is that they are an inherent component of the food product and may be consumed without having to unpack and discard the container. Consumer desire for safe, convenient, stable, biodegradable packaging has driven interest in edible films (Tulamandi et al. 2016).

Many substances derived from agricultural commodities and food industry wastes have been used as materials for edible films. The main components include proteins, polysaccharides, and lipids, which can be ingested into the film (Jiang et al. 2020). It was also considered that covering fruits and vegetables with preservative chemicals creates a changing environment by forming a semipermeable barrier to carbon dioxide, oxygen, and moisture, delaying senescence and ripening and improving the shelf life of fruits and vegetables (Kocira et al. 2021). Edible films also aid in the creation of a changing environment surrounding fruits, as well as the reduction of weight loss during storage and transportation (Parven et al. 2020). The industries (mainly food and pharmaceutical) have recognised edible packaging as a valuable alternative to conventional packaging mainly due to its reduced waste release and novel applications with improved product stability, quality, safety, and consumer-friendliness (Trajkovska Petkoska et al. 2021).

Polysaccharide films provide a pleasing odour, oxygen, oil barrier properties, structural integrity, and strength. They also offer water-resistance properties. The hydrogen-bonded network topology and low solubility give excellent oxygen barrier properties. By delaying ripening, polysaccharide-based films can improve the coated foods' shelf life (Aisyah et al. 2018). Starch is one of the primary materials used in producing edible films. However, starch-based films have several flaws, such as low water resistance and low water vapour barrier, which affect their stability and mechanical capabilities. Starch and arrowroot powder were used to prepare the edible film, particularly in the food and pharmaceutical industries (Alcázar-Alay and Meireles 2015).

Using the second biopolymer in the starch-based composite can be considered an approach for producing materials with relatively low water sensitivity and high strength (Yaradoddi et al. 2022). Using maize, rice starches, various plasticizers, and fillers may substantially influence bioplastics’ physical and chemical characteristics (Krishnamurthy and Ambritkumar 2019; Marichelvam et al. 2019). Vinegar is a 6% acetic acid solution that releases acetate and hydrogen ions. Because hydrogen ions react with starch polymers in solution, they become more disordered. The cast film is more homogenous because of the disorder generated by water interruption and acetic acid ionisation. The film developed with orange peel and glycerol as a plasticizer has good strength, flexibility, and bio-degradability (Yaradoddi et al. 2022). Nano clay incorporation into the starch-based films decreased water uptake (Shafqat et al. 2021). Films prepared using polyethylene glycol as a plasticiser and carrageenan films with different concentrations of arrowroot starch showed various properties at different concentrations. The film with 60% carrageenan showed the best results (Giyatmi et al. 2017). Adding rice starch increases the tensile property and decreases water absorption and solubility (Marichelvam et al. 2019). Increased water vapour permeability, moisture content, thickness, and water solubility were observed when the casting concentration of arrowroot starch in the film generated by casting increased (Fakhouri et al. 2019). The soy protein isolate-based films combined with gelatine and plasticised with glycerol at pH 10 were flexible and transparent. Adding gelatine to the films reduced their hydrophilicity (Chen et al. 2019).

This paper demonstrates the preparation of five edible films using various components such as arrowroot powder, cornstarch, refined wheat flour, glycerol, pectin, and vinegar. Each ingredient used in the film preparation is chosen based on the properties it imparts to the formed film. Because of their ready availability, low cost, biocompatibility, good mechanical strength, transparency, and non-toxic nature, starch-based materials such as cornstarch, wheat flour, and arrowroot are the most promising materials for the production of edible films (Domene-López et al. 2019; Nogueira et al. 2018). Arrowroot starch has a significant amylose content (35.20%), making it ideal for film preparation (Nogueira et al. 2018). Wheat flour is one of the most common sources used as thermoplastic materials for food packaging, primarily containing starch (78–82%) and gluten (8–16%) (Wang et al. 2022). Pectin has received much attention among edible film materials because of its non-toxic, odourless, renewable, oil/fat resistant, and biodegradability. Pectin films have low gas permeability and, thus, good barrier properties (Bermúdez-Oria et al. 2019; Sucheta et al. 2019). As a hydrophilic plasticiser, glycerol improves the film flexibility and mechanical properties of the edible starch film (Youssef and El-sayed 2018). Vinegar is a popular acidic condiment with antioxidant and antimicrobial properties. As a result, it can extend product shelf life and reduce the risk of pathogen growth on food surfaces (Zhang et al. 2018). The physical and mechanical properties of the prepared films were studied along with their characterisation by FTIR and SEM analysis.

## Materials and methods

### Materials

Arrowroot powder, pectin, cornstarch, refined wheat flour (HIMEDIA), vinegar, and glycerol (Bangalore Fine Chemicals) were used in the present work.

### Film preparation

The composition of each film includes Film A (6.5 g Arrowroot powder + 1 g Pectin + 2.5 g Glycerol); Film B (3.5 g Arrowroot powder + 3.5 g Cornstarch + 0.5 g vinegar + 2.5 g Glycerol); Film C (3.5 g Arrowroot powder + 3.5 g refined wheat flour + 0.5 g vinegar + 2.5 g Glycerol); Film D (3.5 g Arrowroot powder + 2 g refined wheat flour + 2 g Cornstrach + 2.5 g Glycerol) and Film E (3.5 g refined wheat flour + 3.5 g Cornstrach + 2.5 g Glycerol + 0.5 g vinegar).

Each composition of film materials was mixed in 100 ml water and stirred under 600 rpm using a magnetic stirrer for about 10 min at 100 °C. Then increased, the temperature was to 120 °C to form gelation and continued stirring for about 70 min at 200 rpm to avoid creating air bubbles. Then it was slowly poured into a Petri dish to form a thin layer and dried carefully in a hot air oven for about 24 h at 40 °C. It was cooled for 3 h. Films are peeled off from the Petri dish carefully. The film samples were stored in a desiccator with silica gel and kept in a humidity digital controller (50% RH) for 24 h before characterisation and measurement of properties.

### Measurement of film properties

#### Thickness of film

The thickness of the films was measured using a thickness gauge (547-500S, Mitutoyo Digital Thickness Gauge, Japan) as per the ASTM D6988 standard. The thickness of each film was measured at five different places on the surface. The average thickness value was calculated using Eq. (1) (Maslahah et al. 2021). This procedure was repeated three times to get accurate and reproducible results

1 $$Thickness=\frac{Sum\, of\, thickness\, values\, obtained\, at\, five\, places}{5}$$

#### Transparency

Transparency is a material's property that indicates the clarity level marked by its ability to transmit light. The film transparency was measured by exposing the rectangular film to light absorption at 550 nm wavelength using a UV (Ultraviolet) spectrophotometer (1800 Shimadzu, Japan). Transparency (*T*) was calculated using Eq. (2) (da Silva et al. 2020).

2 $$T=\frac{ {A}_{550}}{\mathrm{x}}$$

A_550_ indicates the absorbance at 550 nm, and x is the film thickness (mm).

#### Bursting strength, tensile strength, elongation at break, and Young’s modulus

The film's bursting strength was determined using a bursting strength tester (Pack Test Model: 3125 AM, India) as per the TAPPI T403 method. The specimen was fixed between annular clamps, and the hydraulic pressure increased at a stretch until the sample burst using a rubber diaphragm. The sample collapses at a fraction of a second, and the applied hydrostatic pressure corresponding to the bursting point was recorded in the system as the bursting strength.

Tensile strength means the maximum tensile stress the film can sustain, while elongation is the maximum change in length of a film specimen before breaking. A universal testing machine evaluated the tensile strength and elongation at break (Pack Test GP 10, India). Tensile strength indicates the maximum applied force that can withstand until the film can remain stable before breaking. Elongation is the percentage of length increment of a film, measured from the initial length before the applied force and the final length at the withdrawal of the applied pressure until the film breaks. Each film size 15 cm × 0.5 cm was placed between the machine's grips. The tensile strength and elongation values were calculated using Eqs. (3) and (4), respectively (Ahmad et al. 2012; Nawab et al. 2017).

3 $$\mathrm{Tensile\, strength}= \frac{\mathrm{Force}}{\mathrm{Surface\, area}}$$

4 $$\mathrm{Elongation}=\frac{\mathrm{Difference\, between\, initial\, and\, final \,length}}{\mathrm{Initial\, length}}\times 100\%$$

Young’s modulus of the film material indicates the ease with which it can be stretched and deformed. Young’s modulus of the film material is related to its tensile strength and elongation, as represented by Eq. (5).

5 $${\mathrm{Young}}^{\mathrm{^{\prime}}}\mathrm{s \,modulus}= \frac{\mathrm{Tensile\, strength}}{\mathrm{Elongation}}$$

#### Moisture content, water solubility and water vapour permeability

The moisture content could be obtained by checking the weight loss of the film as per the ASTM D4442 method. The film samples were cut into square sizes of 2.0 cm^2^. Initially, the prepared specimen samples were accurately weighed (W_1_). Then, the specimen samples were dried in an oven (Orion DX-708 digital hot air oven) at 100 °C to get a constant weight, and the final weight of the specimen was recorded (W_2_). This procedure was repeated three times, and mean values were used for calculation. The moisture content of the films was obtained by Eq. (6).

6 $${\text{Moisture}}\; {\text{Content}} \left( \% \right) = \frac{{\left( {W_{1} {-} W_{2} } \right)}}{{W_{2} }} \times 100$$

W_1_ is the starting weight, and W_2_ is the final weight of the specimen samples (Marichelvam et al. 2019; Dash et al. 2019).

Three specimen samples (2 cm diameter) of each film were dehydrated at 45 °C for 24 h in an oven to test the film’s water solubility. The initial dry weight (W_3_) of each specimen sample was noted. Then, the specimen samples were submerged in 30 mL of distilled water in a beaker for 24 h at room temperature (25 °C), with occasional stirring. After 24 h, the specimen samples were removed from the water, dried in an oven at 45 °C for 24 h, and weighed (W_4_). The difference in the weights corresponds to the soluble content of the specimen sample. The water solubility of the specimen samples was calculated using Eq. (7) (Abotbina et al. 2021).

7 $${\text{Water}} \;{\text{Solubility}} \left( \% \right) = \frac{{\left( {W_{3} {-}W_{4} } \right)}}{{W_{4} }} \times 100$$

W_3_ and W_4_ represent the initial and final weights of the specimen samples**.**

The water vapour permeability of the films was determined using a circular test cup (No. 318 Water Permeability Cup) by following JISZ 0208 method. The film was cut into a circle slightly larger than the inner diameter of the cup for the test. Anhydrous calcium chloride, a desiccant with 0% relative humidity, was put inside the cup, which was set on a horizontal platform. The permeability cup was then covered with edible film on the top and sealed tightly with liquid paraffin. After measuring the initial weight, the cup was put in a desiccator with a saturated salt solution of magnesium chloride. The edible films’ water permeability properties were studied at room temperature (30 °C). Every 24 h, the cup’s weight was measured until the weight changes were under 10%. The water vapour transmission rate (WVTR) and water vapour permeability (WVP) were calculated using Eqs. (8) and (9) (Othman et al. 2017):

8 $$WVTR=\frac{\Delta m}{\Delta t A}$$

9 $$WVP=\mathrm{WVTR }\left(\frac{L}{\Delta p}\right)$$

where *∆m/∆t* is the moisture gain weight per time (g/s), *A* is the exposed surface area of the film (m^2^), *L* is the thickness of the film (mm), and *∆p* is the difference in partial pressure.

#### Biodegradability

Biodegradability measures the film's resistance to degrading microorganisms, soil, moisture, temperature, and other physicochemical factors. Biodegradability tests were conducted using the weight-loss method. Each film sample (4 cm × 4 cm size) was buried in a plastic jar at around 12 cm depth in the soil and maintained at room temperature for 30 days. The recovered test samples were rinsed under running water to remove the soil residue from the surface and dried at 80 °C in an oven. The initial weight (W_5_) and final weight (W6) before and after testing were recorded. The weight loss obtained after the testing relates to each film sample's biodegradability level. The biodegradation value of edible films was calculated using Eq. (8)

10 $${\text{Weight}}\; {\text{loss}} = \frac{{\left( {W_{5} {-} W_{6} } \right)}}{{W_{6} }} \times 100\%$$

W_5_ represents the initial weight of the specimen sample before the test, and W_6_ is the weight after the test (Marichelvam et al. 2019).

### Films surface study

The surface morphology of the films was taken using a Carl Zeiss EVO 18 analytical scanning electron microscope (SEM) with high acceleration voltage. The film samples were subjected to gold sputtering to avoid unwanted charges during scanning. The shattered film surface was subjected to SEM investigation.

### FTIR spectral study

Each film's FTIR (Fourier transfer infrared) characterization was carried out using Shimadzu IR Sprit, QATR-S FTIR spectrophotometer. IR spectra of constituent materials and the respective films were taken and compared.

### Thermogravimetric analysis (TGA)

The SHIMADZU DTG-60 equipment from Japan was used to perform TGA. The weighed samples (5.0–8.0 mg) were put into aluminium pans and then subjected to a dry nitrogen atmosphere (60 mL/min) heated from 30 to 500 °C at 10 °C per minute.

### Statistical analysis

An analysis of variance (ANOVA) is performed using Python to analyse the significance of the measured properties of films. The probability value (*p*) of less than 0.05 was used as the criterion to conclude the significant differences in the properties of edible film. A post hoc Tukey HSD test compared film properties pairwise as a follow-up to ANOVA.

## Results and discussion

### The film properties

The thickness of all films was calculated, and the average thickness values are shown in Table 1. The packaging films must be at least 0.05 mm thick, according to Indian government standards (Marichelvam et al. 2019). The prepared edible films have a thickness in the 0.159–0.384 mm range (*p* ˂ 0.05; Table 1), indicating their suitability for packaging. Similar results were obtained in edible films based on native cassava starch with a thickness of 0.22–0.44 mm (Silva et al. 2019). Generally, the thickness of the edible film is less than 0.33 mm (Ayquipa‑Cuellar et al. 2021). A considerable amount of glycerol in all the film formulations might have increased the thickness of all the films due to the increase in the viscosity of the formulation solution (Aisyah et al. 2018). The thickness of the film is an important parameter that influences both mechanical properties and water vapour permeability (Shaikh et al. 2018).

**Table 1:** Thickness and transparency of films

Film	Thickness* (mm)	A_550_	Transparency*
A	0.180 ± 0.001	0.284	1.578 ± 0.013
B	0.261 ± 0.002	0.213	0.816 ± 0.007
C	0.384 ± 0.003	0.629	1.638 ± 0.014
D	0.183 ± 0.001	0.464	2.535 ± 0.022
E	0.159 ± 0.001	0.427	2.685 ± 0.026

*Mean value ± standard deviation (n = 3)

Transparency is a film's nature to show clarity based on its ability to transmit light. The transparency values of the films varied from 0.816 to 2.685 (*p* ˂ 0.05; Table 1). Films D and E showed comparatively higher transparency, while film B exhibited less. It was found that all prepared films showed a higher absorption value in the UV range (200–400 nm) and a lower absorption value in the visible range (400–800 nm) (Fig. S1 in Supplementary material). Due to the high absorption level observed in the UV range, all the films could provide an excellent barrier against UV light-inducing lipid oxidation when employed in food packaging (Nawab et al. 2017; Florez et al. 2022). The reported edible films based on mucilage from *Opuntia ficus indica* showed the highest transparency, 3.82–7.43 (Sandoval et al. 2019), whereas the prickly pear peel mucilage and potato husk starch edible films exhibited the lowest transparency, 0.089–0.541 (Ayquipa‑Cuellar et al. 2021).

Poor flexibility or strength can lead to premature failure or cracking during production, handling, storage, or use, so edible films must have good mechanical properties. (Kocira et al. 2021). The bursting strength measures the maximum weight or pressure the film material can withstand before breaking. The estimated bursting strength of the edible films varies in the range of 1.5–2.3 kg/cm^2^ (*p* ˂ 0.05; Table 2). It is evident (Table 2) that Film B exhibited the highest bursting strength; however, Films A and E showed the least. The tensile strength (0.12–0.14 kg/ cm^2^), elongation at break (35–46%), and Young’s modulus (0.0031–0.0037 kg/ cm^2^) of all the films (*p* ˂ 0.05) are tabulated in Table 2. The presented results indicate that Film B shows the maximum tensile strength and elongation at break. Films B, C, and E with vinegar content have offered higher tensile strength and elongation. The higher the elongation at break, the better the film quality with good tensile strength. The film with less than 5% elongation at break is said to be brittle. However, all the films showed elongation at break well above 5%, showing flexible nature. The films with lower elongation at break usually offer a higher Young's modulus (Film B). Pectin films showed lower tensile strength (0.036–0.099 kg/cm^2^) and elongation at break (4.9–12.4%) (Galus et al. 2013). Biodegradable films based on methylcellulose and jambolao (Syzygium cumini) skin extract displayed comparatively lower elongation at break (9.1–37.5%) (da Silva et al. 2020).

**Table 2:** Bursting strength, tensile strength, elongation at break, and Young’s modulus of films

Film	Bursting strength* (kg/cm^2^)	Tensile strength* (kg/cm^2^)	Elongation at break* (%)	Young’s modulus * (kg/cm^2^)
A	1.5 ± 0.013	0.1230 ± 0.001	35.33 ± 0.34	0.0035 ± 0.0002
B	2.3 ± 0.021	0.1427 ± 0.001	46.00 ± 0.44	0.0031 ± 0.0001
C	1.8 ± 0.016	0.1399 ± 0.001	40.66 ± 0.40	0.0034 ± 0.0001
D	1.8 ± 0.017	0.1295 ± 0.001	38.00 ± 0.37	0.0034 ± 0.0002
E	1.6 ± 0.015	0.1324 ± 0.001	36.00 ± 0.35	0.0037 ± 0.0002

*Mean value ± standard deviation (n = 3)

The moisture content obtained for the prepared films varies from 6 to 16% (*p* ˂ 0.05; Table 3). The results show that films D, C, and B exhibit lower moisture content while films E and A exhibit a higher value. Thus, films B, C, and D show the best results. The water solubility of the edible films lies in the range of 23–35% (*p* ˂ 0.05; Table 3). The results indicate that film B shows the minimum water solubility. However, films A and E revealed higher water solubility. Starch granules and arrowroot content show less water solubility at room temperature (Alcázar-Alay and Meireles 2015). The glycerol might be responsible for the increasing water solubility due to its hydrophilic nature. They are crucial in weakening the connections between polymer molecule chains, resulting in more free space between chains. It allows water to seep into the polymer matrix, increasing solubility (Shafqat et al. 2021). A similar range of moisture content (10–12%) and higher water solubility range (39–54%) were reported on prickly pear peel mucilage and potato husk starch edible films (Ayquipa‑Cuellar et al. 2021). Methylcellulose and jambolao (*Syzygium cumini*) skin extract-based films showed high water solubility of 100% (da Silva et al. 2020). Mango kernel starch-gum composite films showed higher water solubility (36–44%) (Nawab et al. 2017). Saberi et al. (2016) reported higher water solubility (29%) and moisture content (20%) for an edible film based on pea starch and guar gum.

**Table 3:** Moisture content, water solubility, water vapour permeability, and biodegradability of films

Film	Moisture content* (%)	Water solubility* (%)	WVP* (g.mm/(m^2^.day.mmHg))	Biodegradability* (%)
A	13.69 ± 0.12	33.33 ± 0.32	0.0019 ± 0.0001	54.28 ± 0.48
B	8.51 ± 0.07	23.25 ± 0.23	0.0035 ± 0.0001	73.66 ± 0.68
C	7.52 ± 0.07	27.32 ± 0.26	0.0113 ± 0.0005	91.33 ± 0.82
D	6.76 ± 0.06	24.35 ± 0.22	0.0106 ± 0.0004	70.53 ± 0.64
E	16.39 ± 0.16	35.29 ± 0.34	0.0021 ± 0.0001	60.65 ± 0.52

*Mean value ± standard deviation (n = 3)

Water is a critical factor in food deterioration, so an essential feature of edible films is their ability to prevent moisture exchange between the environment and the food matrix (Basiak et al. 2017). Water vapour permeability (WVP) is the amount of moisture that passes through a unit of material area per unit of time. Low values indicate that the products have a longer shelf life (Nogueira et al. 2018). In this study, the water vapor transmission rate (WVTR) is calculated by dividing the slope of the regression line of the sample weight versus the time graph by the area of the film exposed to transmission (Eq. (8)). The WVP of the prepared edible films (Eq. (9)) lies in the range 0.0019–0.0035 g.mm/(m^2^.day.mmHg) (*p* < 0.05; Table 3). It can be observed from Table 3 that the WVP of the film A are least followed by films E and B. Films C and D have shown relatively higher WVP. Thus, it can be concluded that film A can offer the highest protection to the product against the penetration of moisture compared to the other films. The literature reported that edible films made from potato peel waste displayed higher WVP, 0.216–0.268 g.mm/(m^2^.day.mmHg) (Othman et al. 2017) compared to the edible films prepared in the present work.

Biodegradation leads to the loss of mechanical properties, fragmentation, or chemical modifications due to the action of microorganisms and enzymes. Biodegradability measures the duration of the complete degradability of the film under soil burial treatment. After 22 days of continuous soil immersion, all samples had partially degraded. Most samples showed colour and shape changes during this time (Susllawati et al. 2019). During 60 days, most of the samples had degraded such that they were indistinguishable from the soil. Table 3 shows the extent of biodegradability after 22 days. Film C offers good biodegradability.

### SEM analysis

**Fig. 1:**
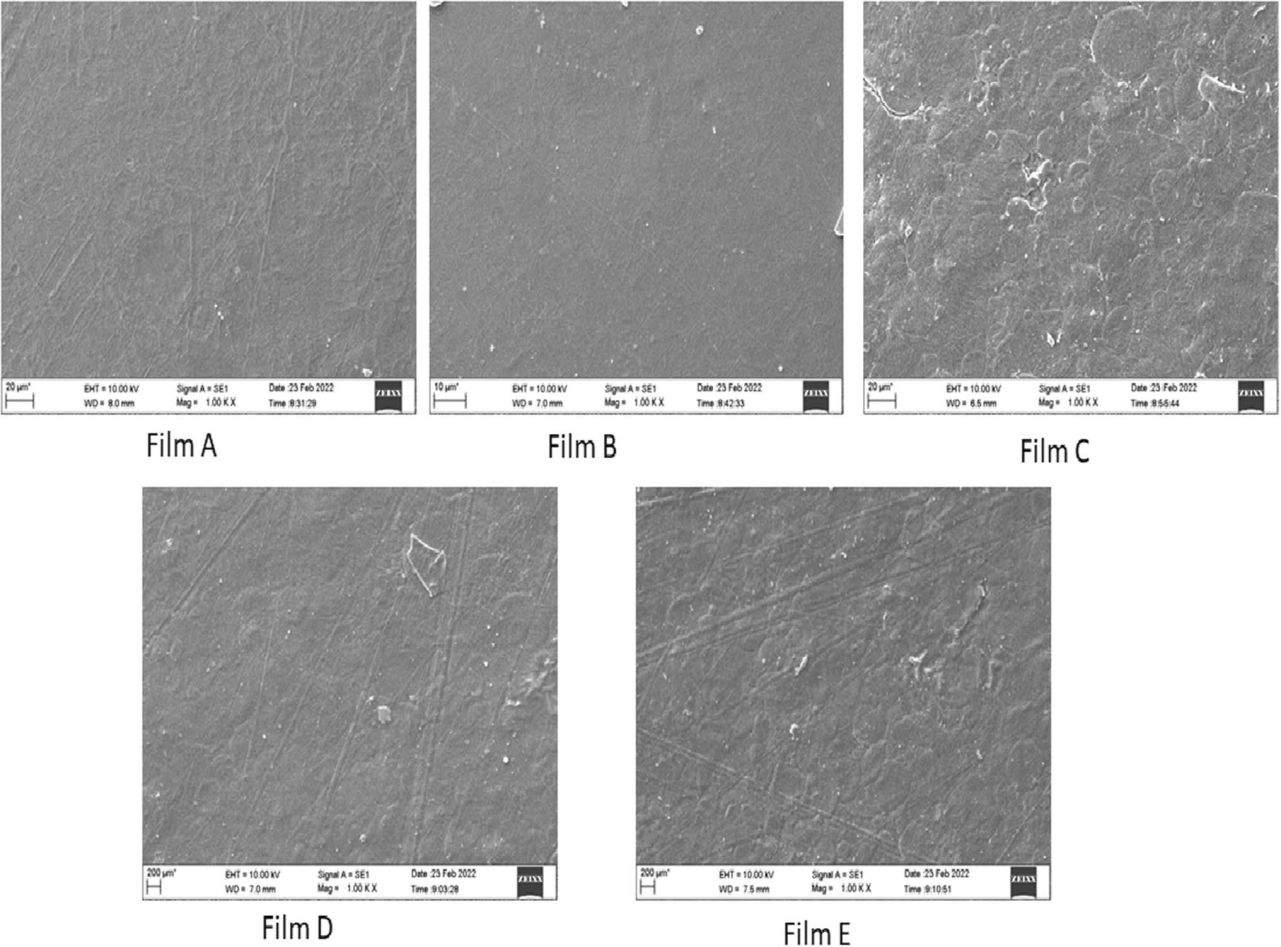
SEM images of edible films A, B, C, D, and E

SEM images of the prepared films are depicted in Fig. 1. The SEM analysis helps to check the films' surface homogeneity, smoothness, and the presence of cracks, which can influence the mechanical properties of films. The films look very smooth; however, there are still little microspores. Some scratches on the films are due to the unevenness of the substrate on which the film was cast. The films with refined wheat flour (C, D, and E) showed surface unevenness due to the larger particle size of wheat flour (Wardak et al. 2021).

### FTIR analysis

**Fig. 2:**
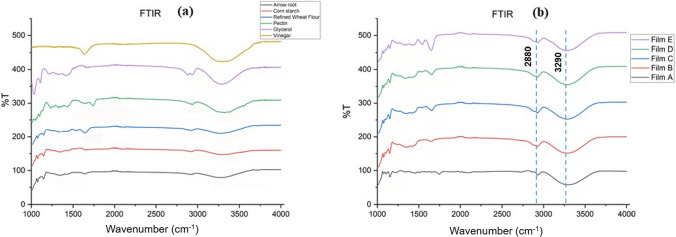
FTIR spectra of **a** arrowroot, corn starch, refined wheat flour, pectin, glycerol, and vinegar and **b** Edible films A, B, C, D, E

FTIR spectra of arrowroot powder, refined wheat flour, cornstarch, glycerol, pectin, and vinegar are shown in Fig. 2a, and FTIR spectra of edible films are shown in Fig. 2b. FTIR results for the edible films revealed no new peaks; hence the edible film’s preparation involves the physical blending of components. The FTIR spectra of the prepared films (Fig. 2b) show an absorption band at 3000–3500 cm^−1^, corresponding to the stretching vibration of the –OH group connected with inter- and intramolecular bonds of the hydroxyl group of nearby starch molecules (Guadarrama-Lezama et al. 2018) revealing the formation of hydrogen bond between the constituent’s materials in each film (Pineros-Hernandez et al. 2017). Hence, hydrogen bonding can play an essential role in film formation and its characteristic properties. The absorption band of the films at 2830–2920 cm^−1^ corresponds to the stretching vibrations of C–H and C–H_2_ bonds of glycerol (Gheribi et al. 2018; Tee et al. 2017).

### TGA results

**Fig. 3:**
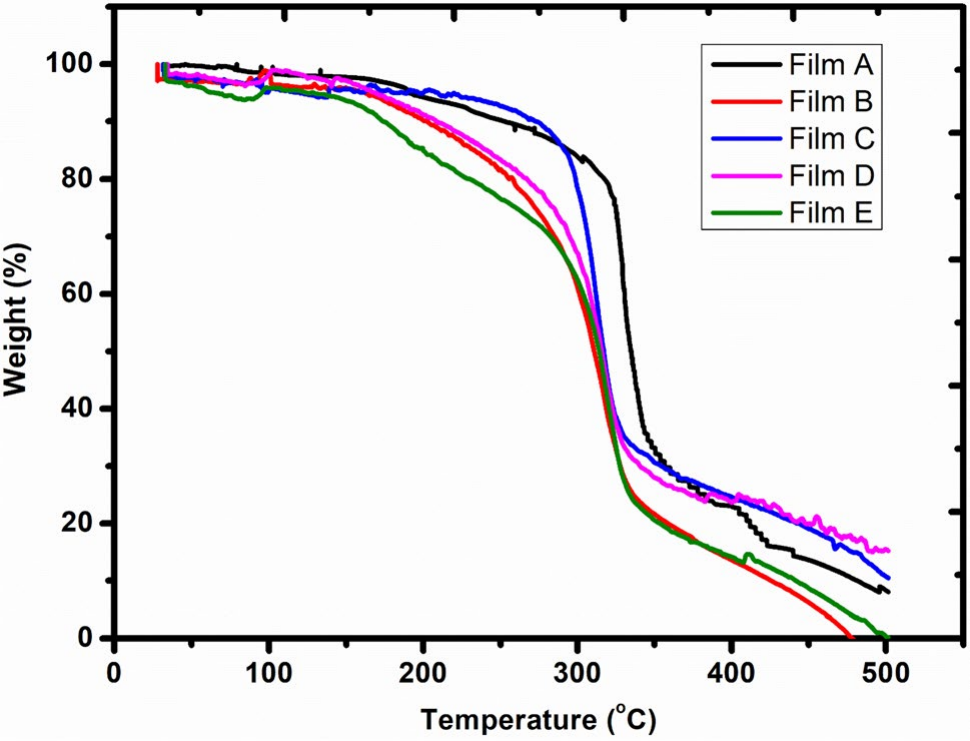
Thermogram of films A, B, C, D, E

Thermogravimetric analysis (TGA) helps evaluate the film material's thermal stability based on its mass variation with temperature. The thermogram of edible films A, B, C, D, and E are depicted in Fig. 3. In the first stage; the films followed weight loss from 2 to 5% in the temperature range 30–100 °C attributed to moisture vaporization (Chu et al. 2019). All the prepared films demonstrated high thermal stability, with a weight retention of about 90–95% up to 190 °C. This reveals that the prepared edible films could be safely subjected to pasteurization treatments. The films showed a weight loss of 6–25%. between 200 and 300 °C temperature. The films A and C revealed higher thermal stability up to 280 °C. The decomposition of bioactive constituents, including arrowroot, corn starch, refined wheat flour, glycerol, and vinegar, led to a significant weight loss between 300 and 400 °C. The decomposition of the glycerol-rich phase caused mass loss around 300 °C, and the partially decomposed starch underwent oxidation at temperatures above 300 °C (Pineros-Hernández et al. 2017).

### ANOVA and post-hoc Tukey HSD test results

The ANOVA results (Table S1 (Supplementary materials) and Table 4) for the five edible film’s measured properties indicate a significance level of 5%. It is observed that the differences in the values of thickness, transparency, tensile strength, elongation at break, Young's modulus, bursting strength, moisture content, water solubility, biodegradability, and WVP properties of edible films are significant as the *p* values are less than 0.05. Hence, it can be concluded that the bursting strength of film B is significantly higher than other films. The moisture content of films D, C and B is considerably lower than films A, and E. It may also be concluded that film C degrades faster, followed by film B when mixed with soil. Film B's water solubility is significantly lower than the other films. The lower degree of water solubility indicates the better resistance of edible films against moisture. It is regarded as one of the most desirable properties in food packaging applications (Tafa et al. 2023).

**Table 4:** ANOVA results for the measured properties of films

Properties	ANOVA result		
	F statistic	*p*	Conclusion
Thickness	13,252	2.07e^−26^	Significant
Transparency	377,087	2.57e^−37^	Significant
Tensile strength	3963	1.76e^−22^	Significant
Elongation	407.18	4.24e^−15^	Significant
Young’s modulus	3.65	0.0285	Significant
Bursting strength	17.89	0.0094	Significant
Moisture content	3015	1.36e^−21^	Significant
Water solubility	51.34	6.26e^−09^	Significant
Biodegradability	22,232	4.28e^−28^	Significant
WVP	4153.53	1.23e^−22^	Significant

Post-hoc Tukey’s HSD test (Table S2 in Supplementary materials) is performed to identify which specific pairwise comparisons between the means of measured film properties are responsible for the overall significant difference observed in ANOVA results. Thickness and WVP of film A and film D were found to be insignificant and shown significant for other pairwise comparisons. Young’s modulus between film B and film E was found to be significant and remained insignificant for other pairwise comparisons. Elongation, tensile strength, transparency, moisture content, and biodegradability properties have shown significant differences for all pairwise comparisons. Bursting strength between films A and B, B and C, B and D, and B and E have shown significant differences while remaining insignificant for other pairwise comparisons. The water solubility between films A and B was insignificant and remained significant for all other pairwise comparisons (Chakravartula et al. 2019).

## Conclusion

Five edible films were prepared with various blends of low-cost materials such as arrowroot powder, corn starch, refined wheat flour, pectin, glycerol, and vinegar. The films B, C, and D showed the highest bursting strength (1.8–2.3 kg/cm^2^), lowest moisture content (6.76–8.51%), and water solubility (23.25–27.32%). All the prepared films have thicknesses well above 0.05 mm, the accepted thickness for packaging films. Film E showed the highest transparency (2.685), while film B exhibited the lowest (0.816). Film B displayed good tensile strength (0.1427 kg/cm^2^) and elongation (46%). A lower value of water vapour permeability of the films (0.0019–0.0035 g.mm/(m^2^.day.mmHg) indicates their suitability for food packaging. All prepared films are partially biodegraded after 22 days; however, complete biodegradation occurs after 60 days. All the edible films exhibited good thermal stability up to 190 °C with a mass loss of about 5%. Films A and C showed higher thermal stability up to 280 °C. The higher thermal stability of the prepared films indicates that they could be safely allowed for pasteurization. SEM analysis revealed that the films (C, D, and E) with refined wheat flour as one of the constituents showed uneven surface texture because of the larger particle size of wheat flour. The FTIR analysis of each constituent with the respective films indicated the absence of any chemical interaction among the components involved. However, the physical interaction, like hydrogen bonding among the constituent materials, cannot be ruled out. The films' observed physical and mechanical properties confirm their suitability as food packaging.

## Supplementary Information

Below is the link to the electronic supplementary material.

Supplementary file1 (DOCX 2834 KB)
