# Supplementary material for: Formulation and characterization of starch-based novel biodegradable edible films for food packaging
Source: J Food Sci Technol. 2023 Aug 19;60(11):2858–67. doi: 10.1007/s13197-023-05803-2 (PMC10497475; doi:10.1007/s13197-023-05803-2)

**Formulation and Characterization of Starch-Based Novel Biodegradable Edible Films for Food Packaging**

**Supplementary Materials**

**
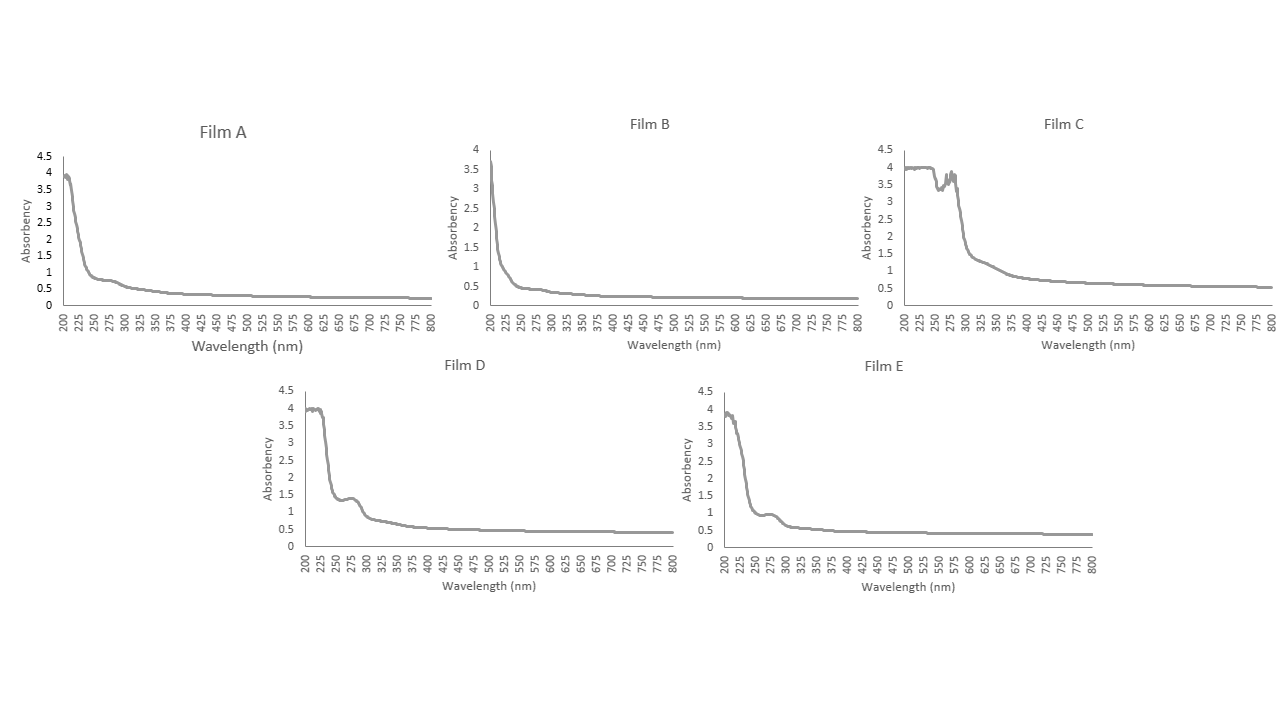
**

**Fig. S1** The graph showing the changes in the absorbency of the films in the 200-800 nm wavelength range

**Table S1: Python codes for one –way ANOVA test**

**Thickness**


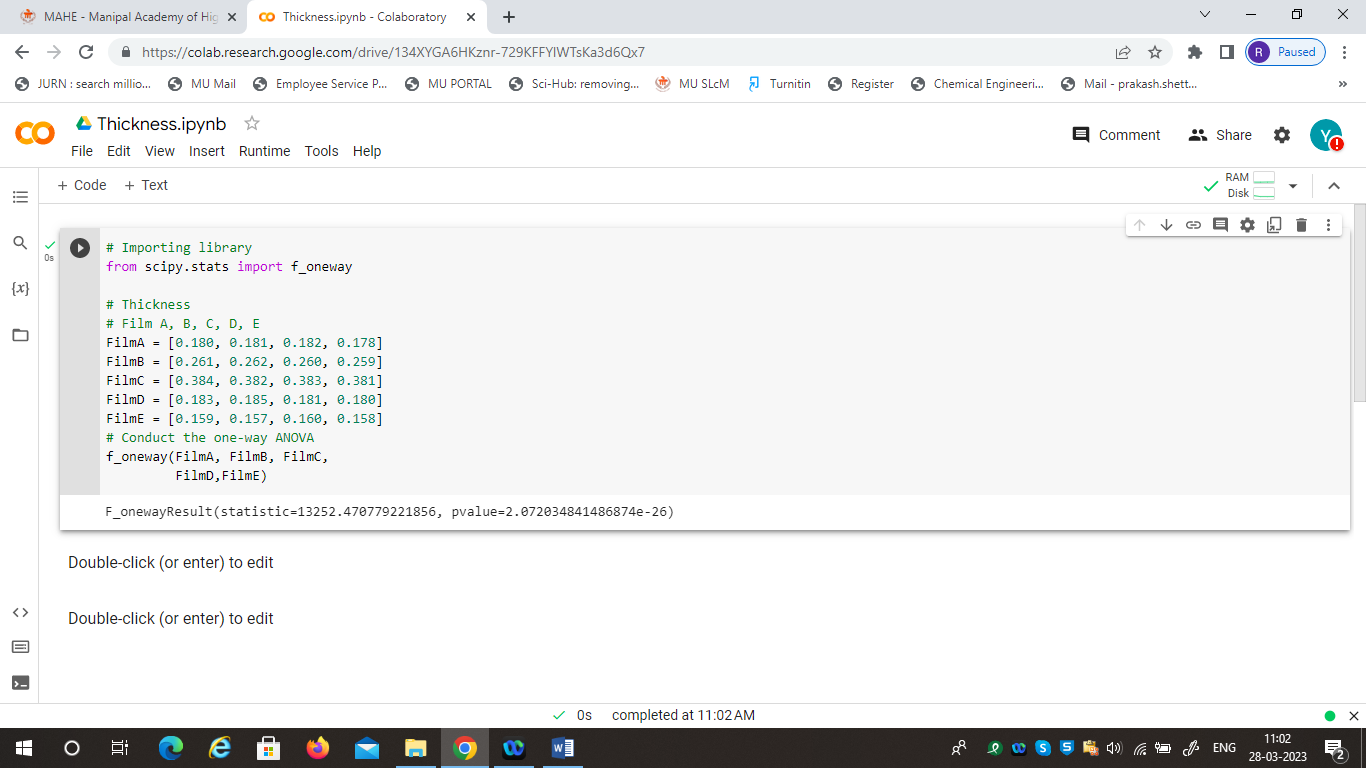


**WVP**


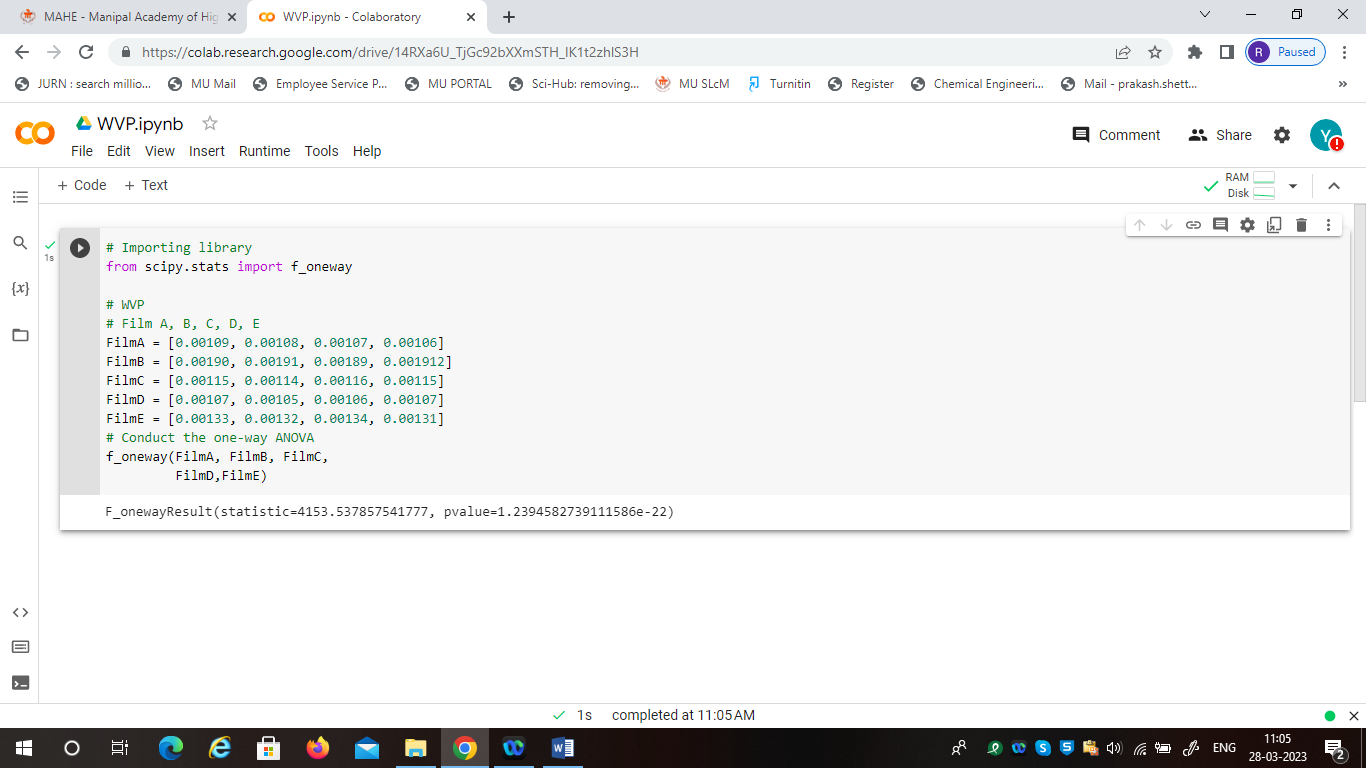


**Young’s modulus**


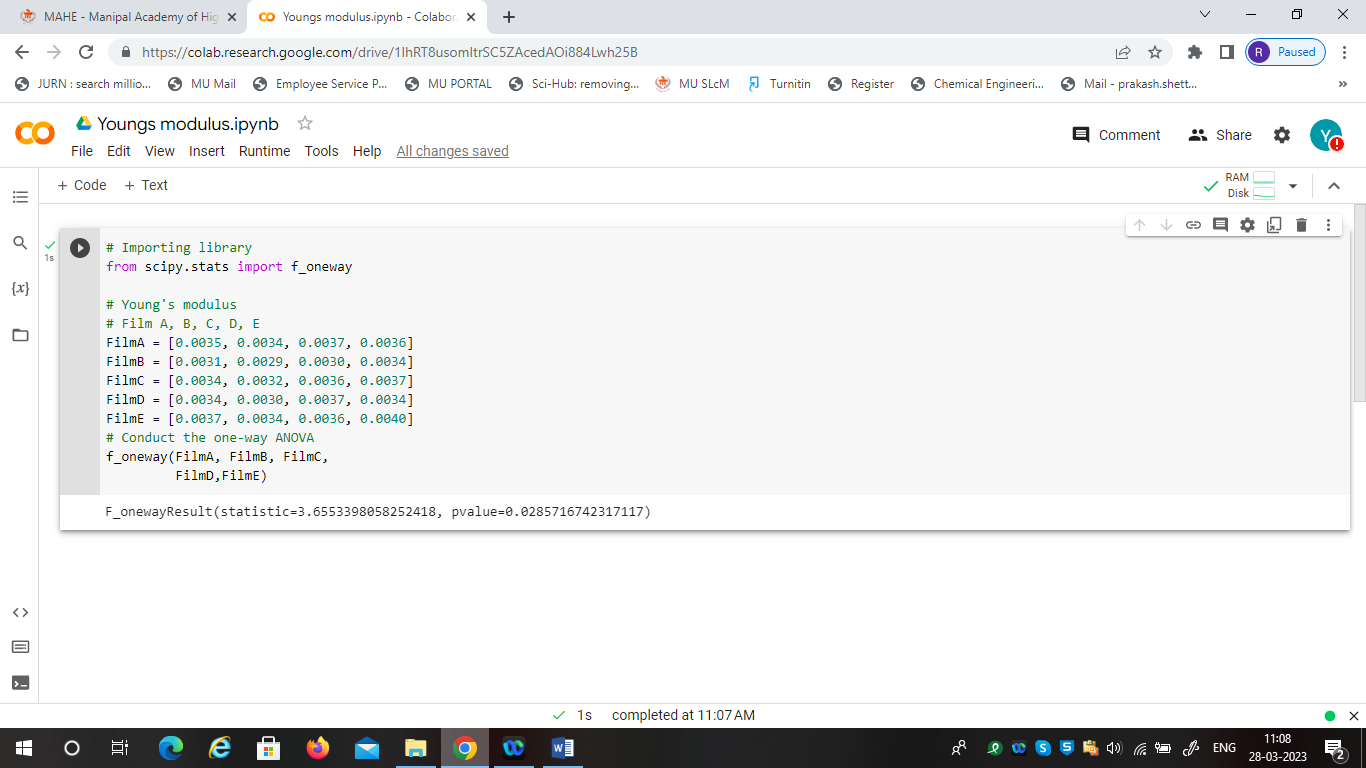


**Elongation**


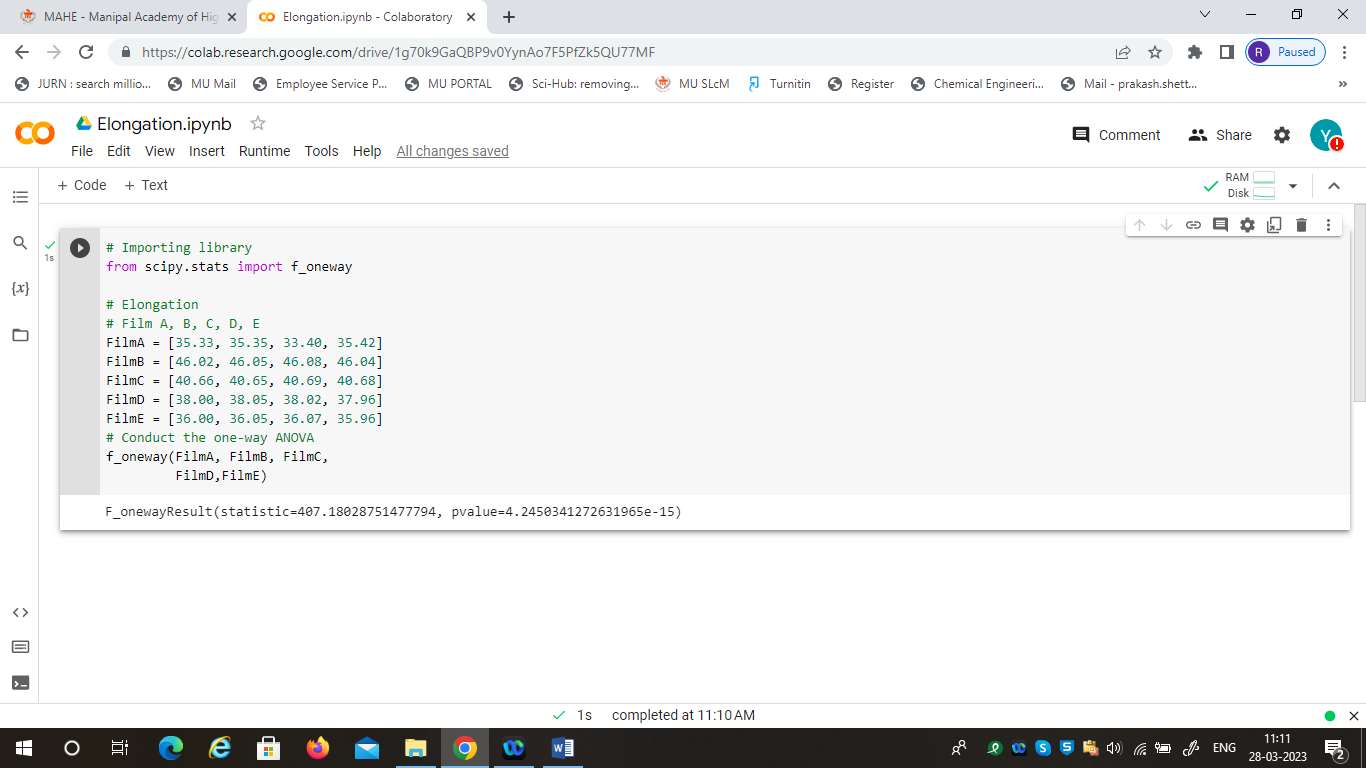


**Tensile Strength**


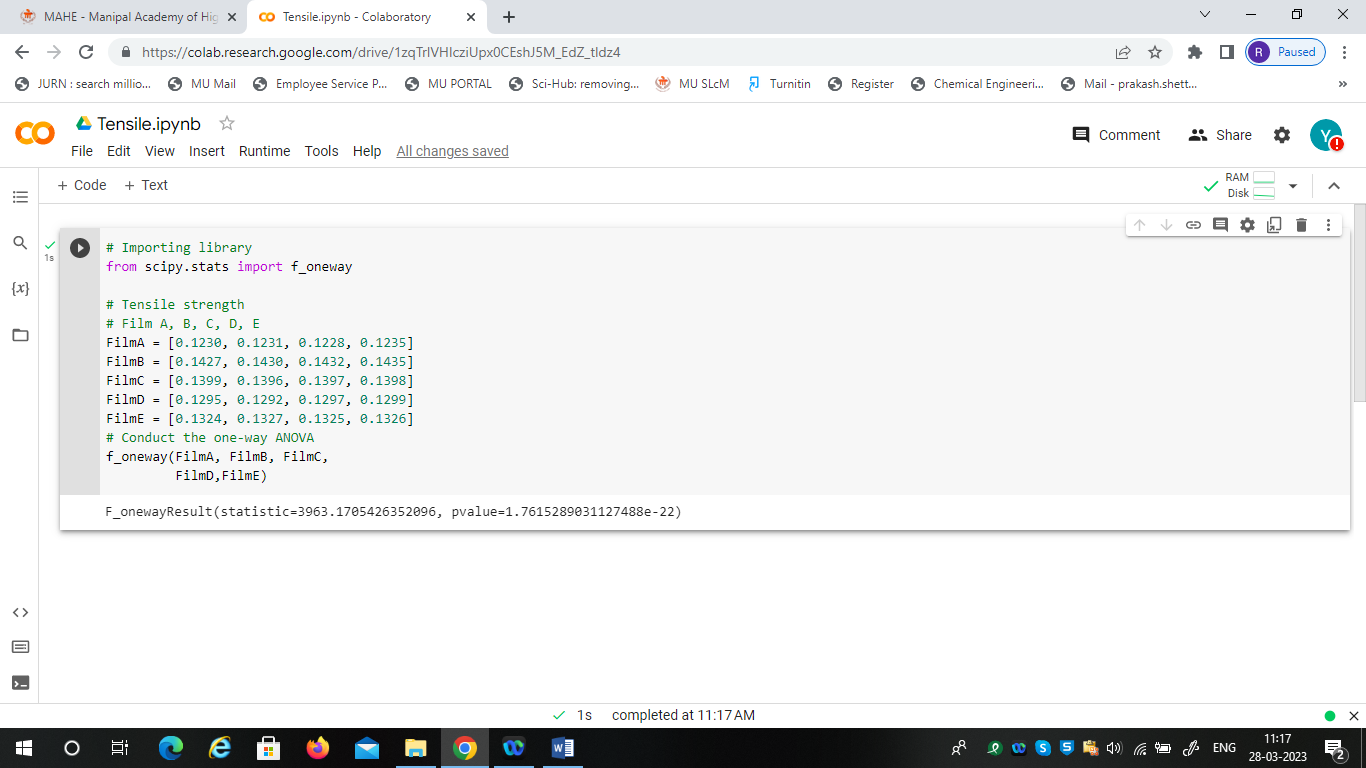


**Transparency**


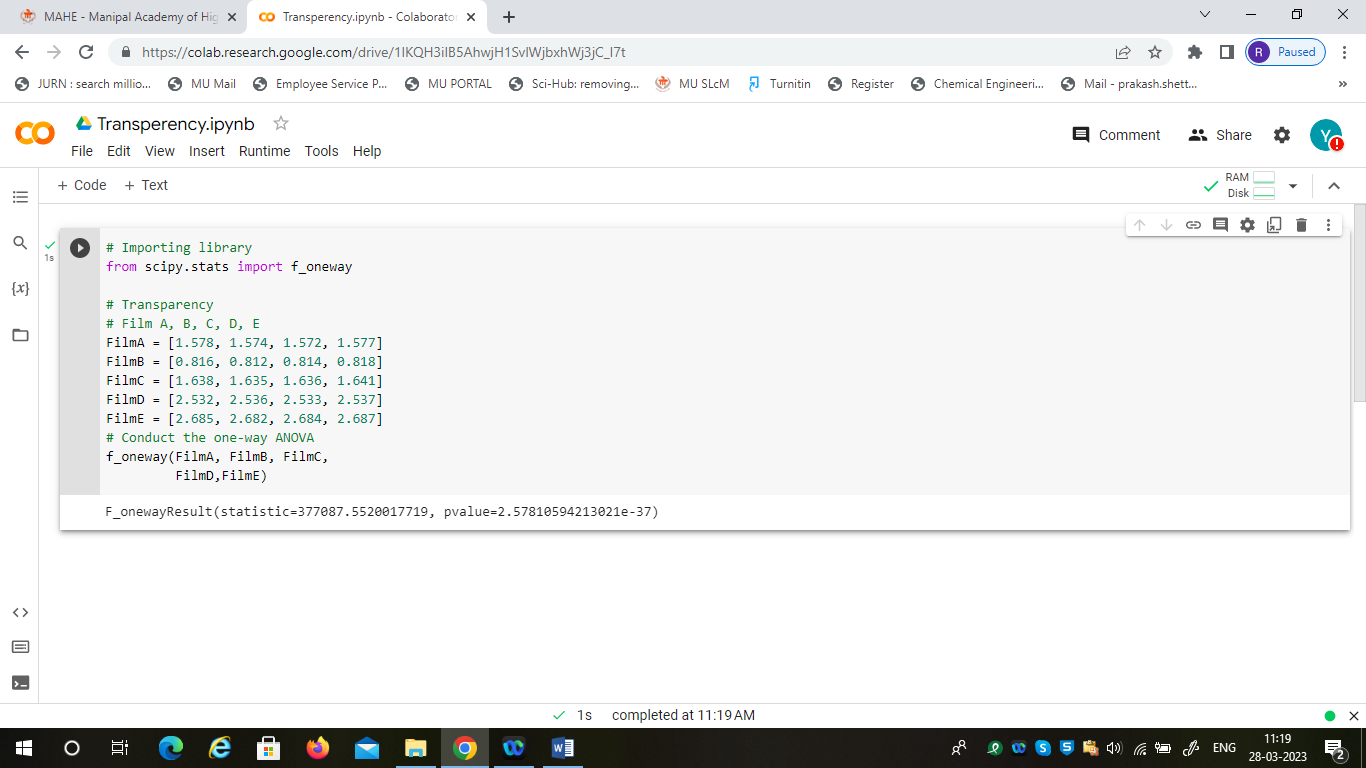


**Bursting strength**


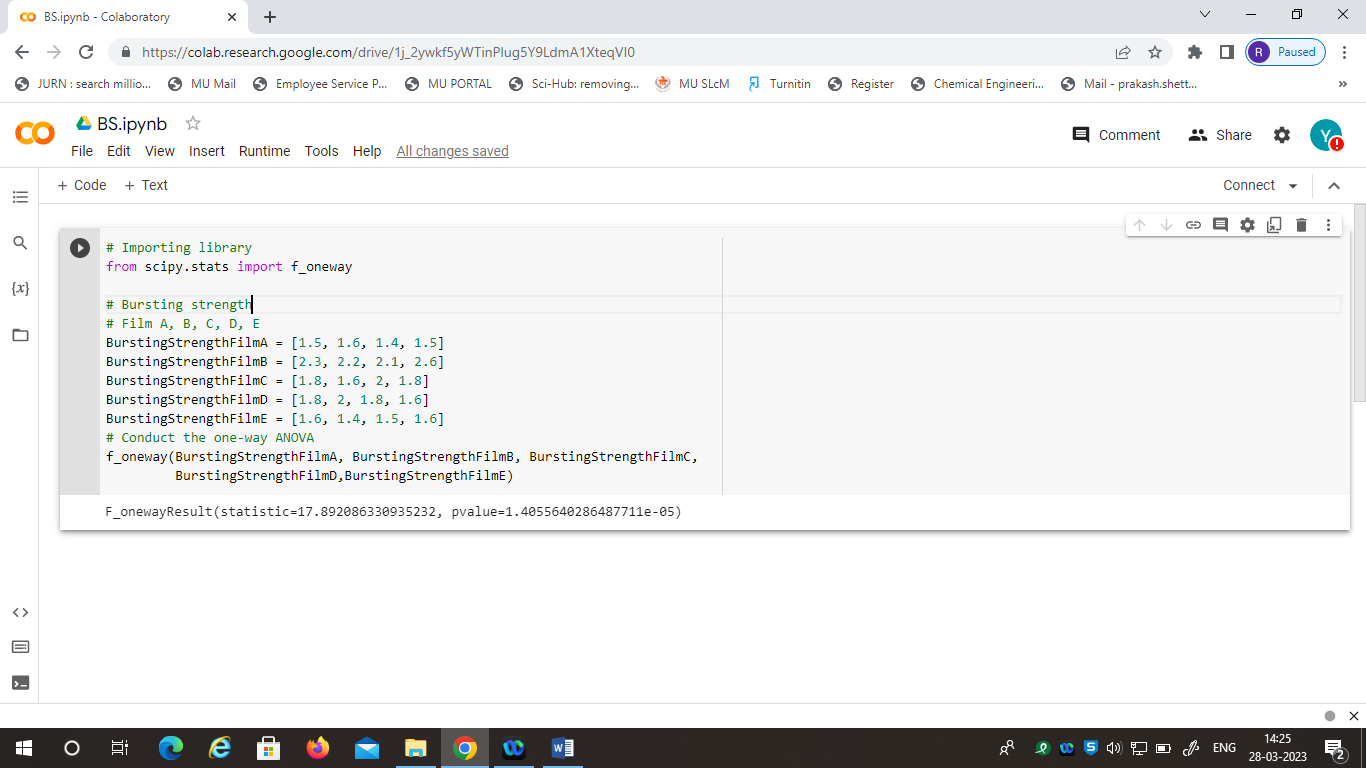


**Moisture content**


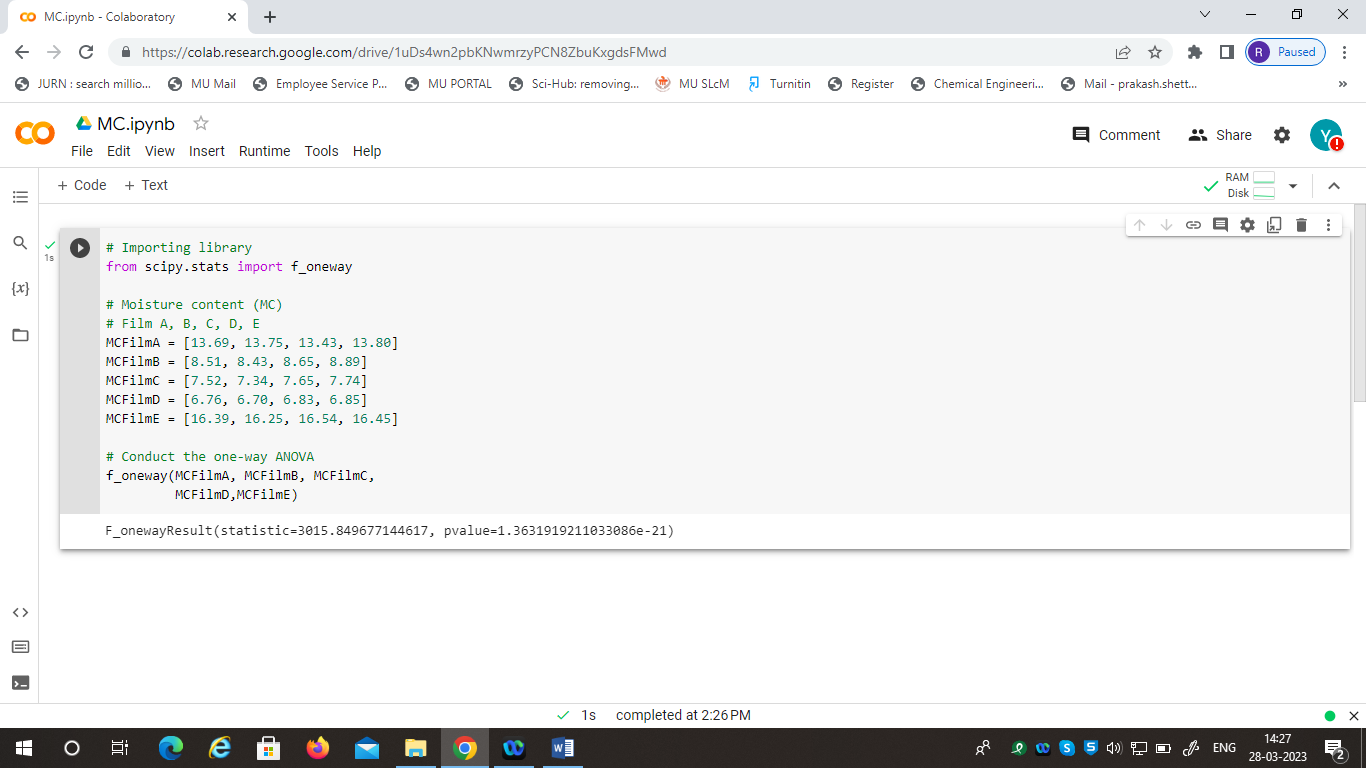


**Water solubility**


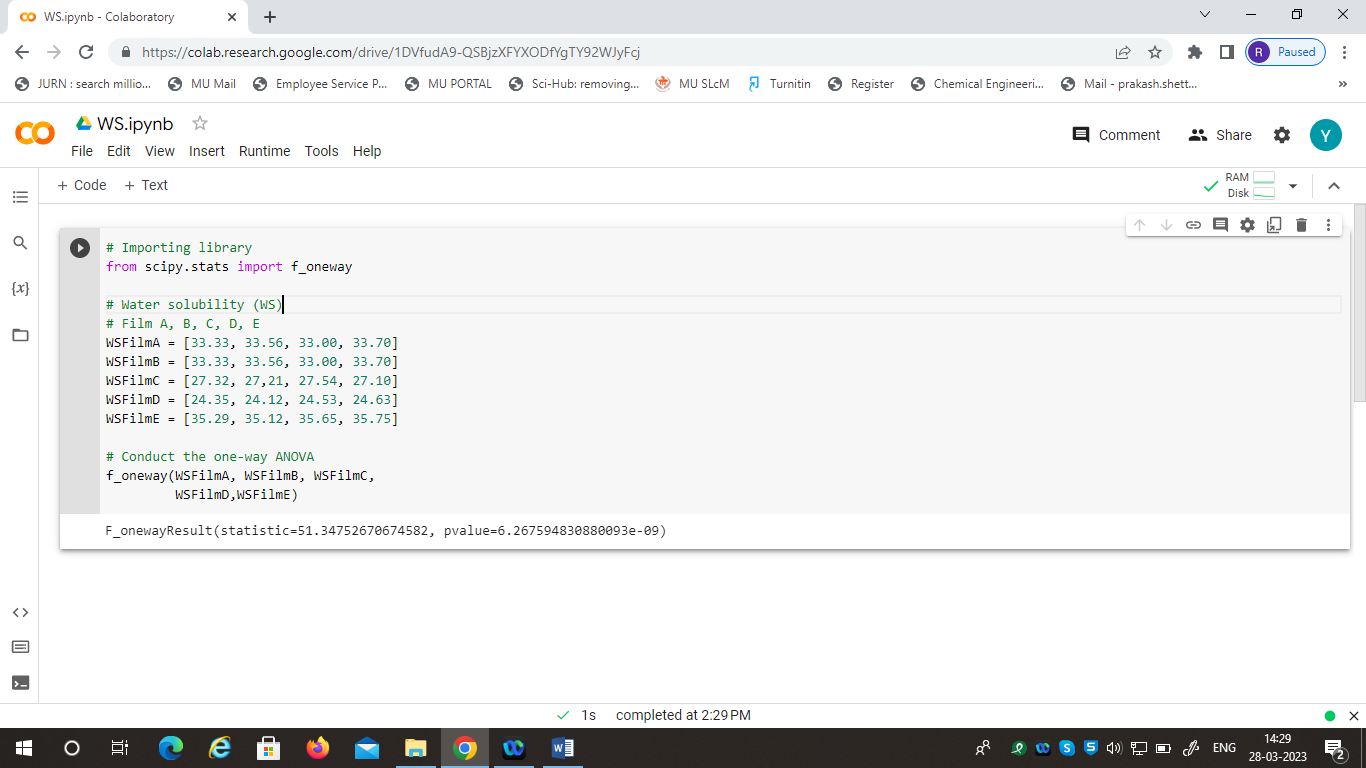


**Biodegradability**


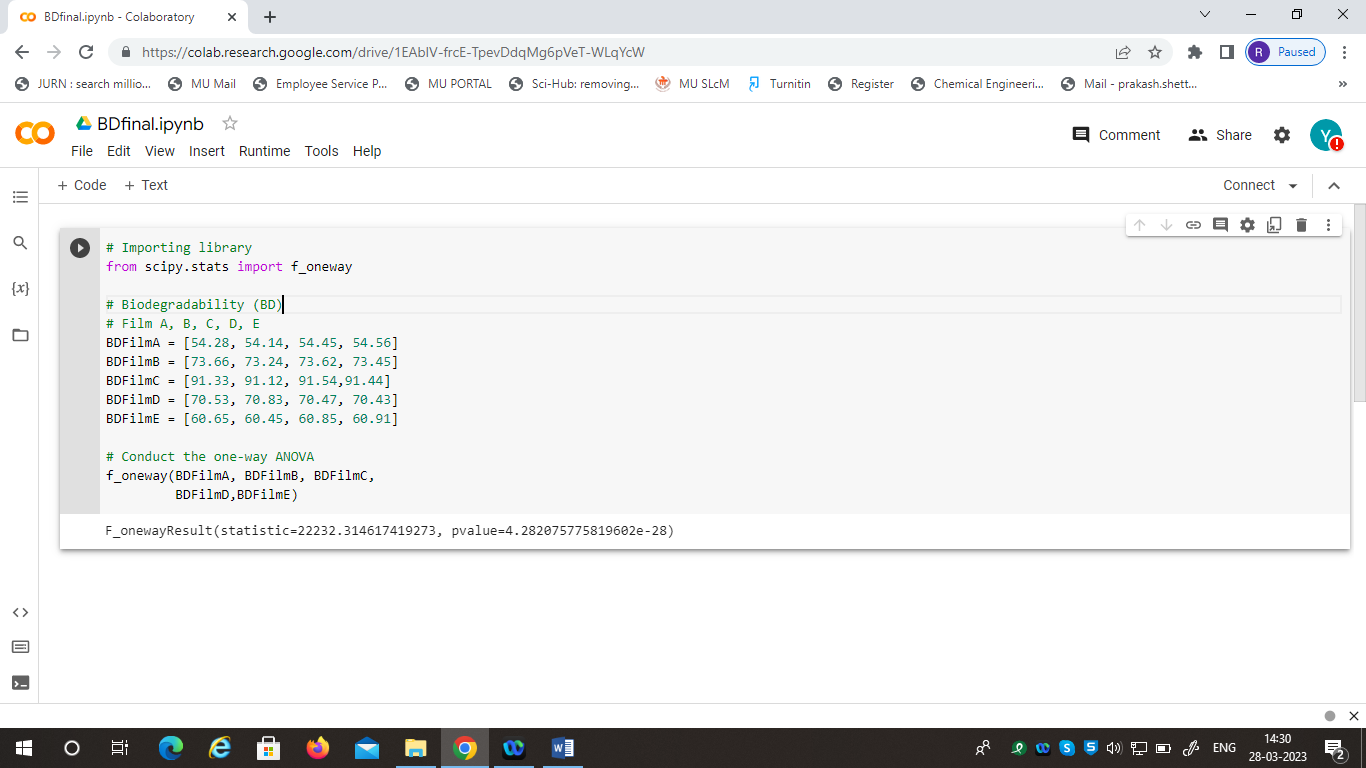


**Table S2: Post-hoc HSD Tukey’s test results**

**Thickness**


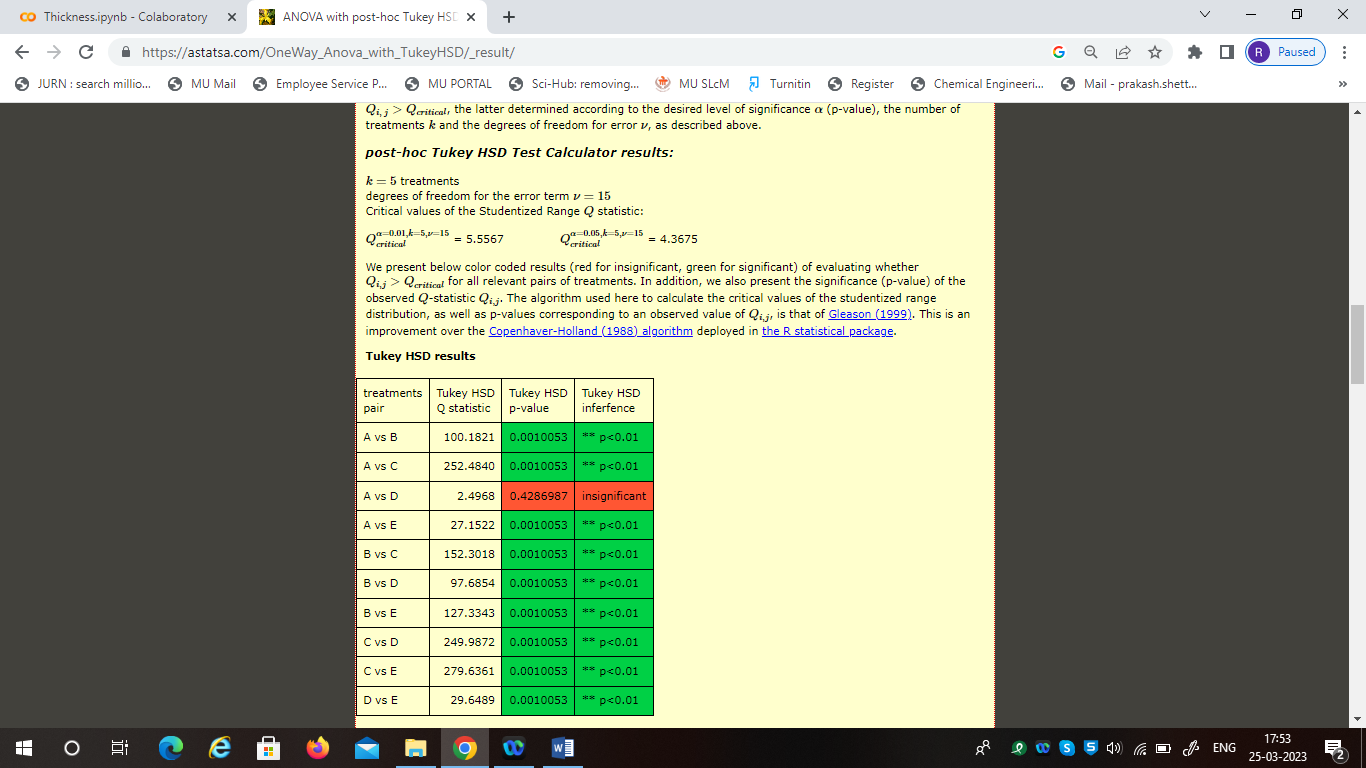


**WVP**


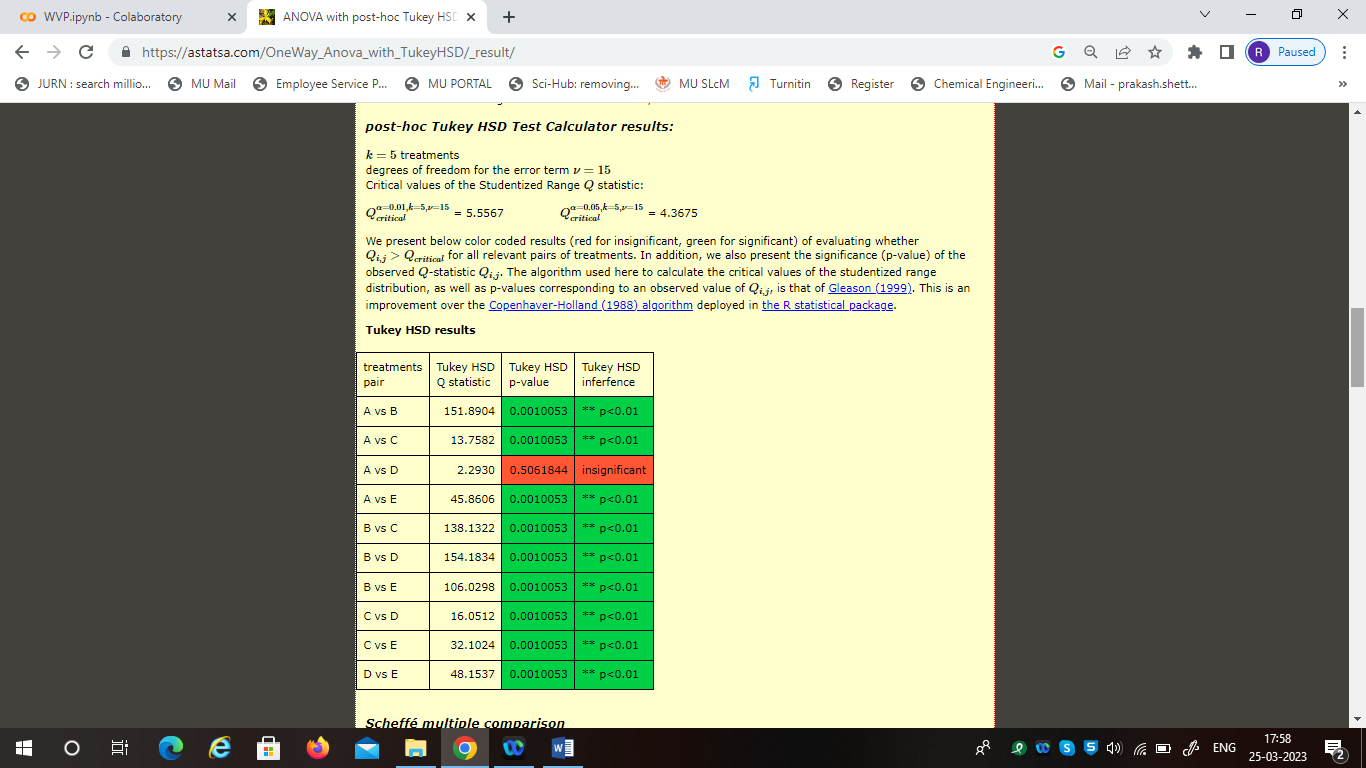


**Young’s modulus**


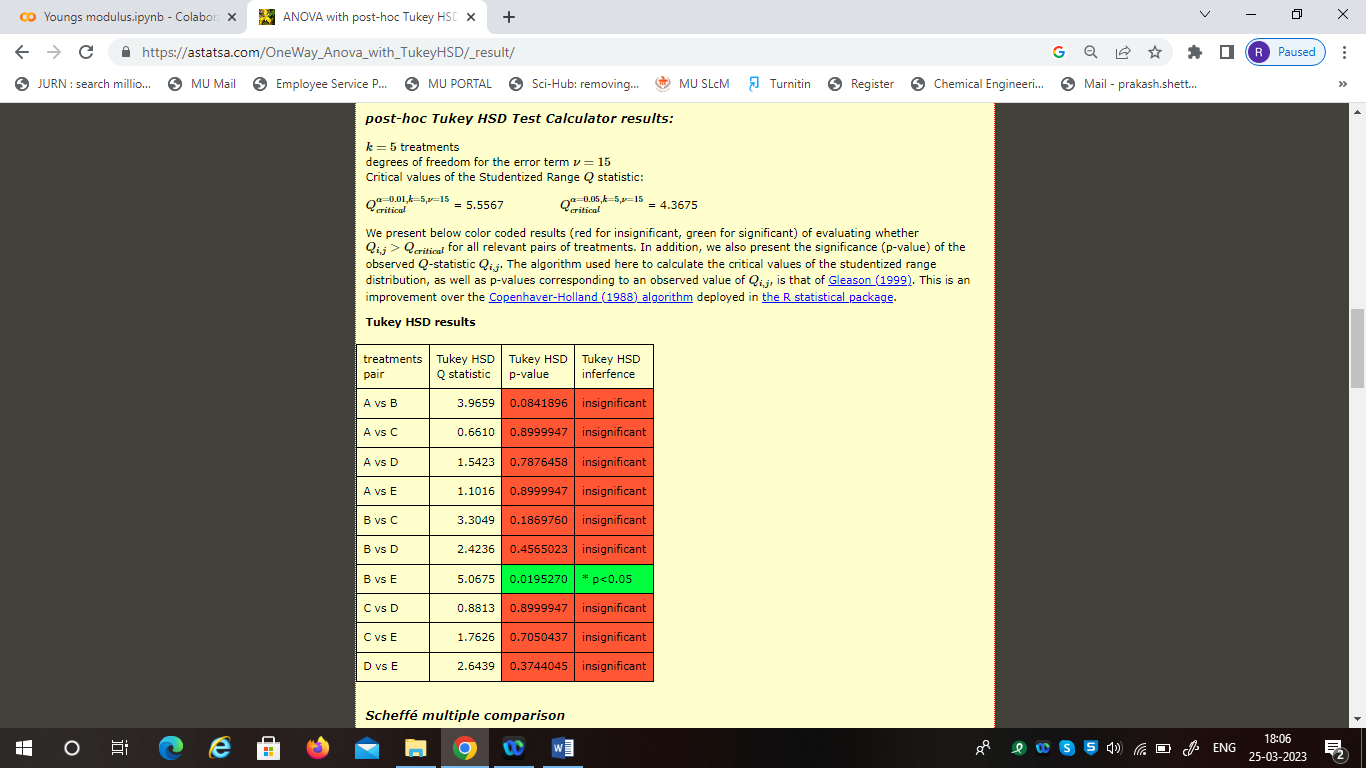


**Elongation**


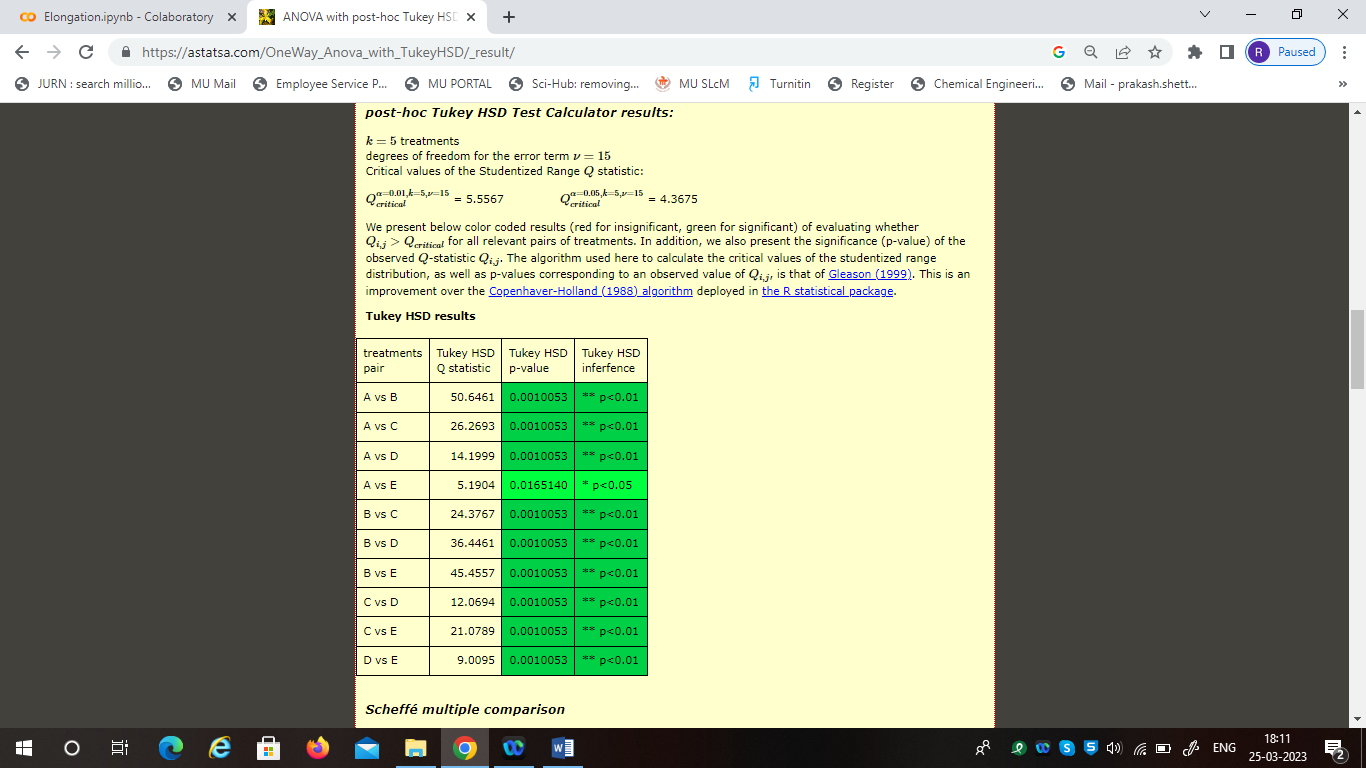


**Tensile Strength**


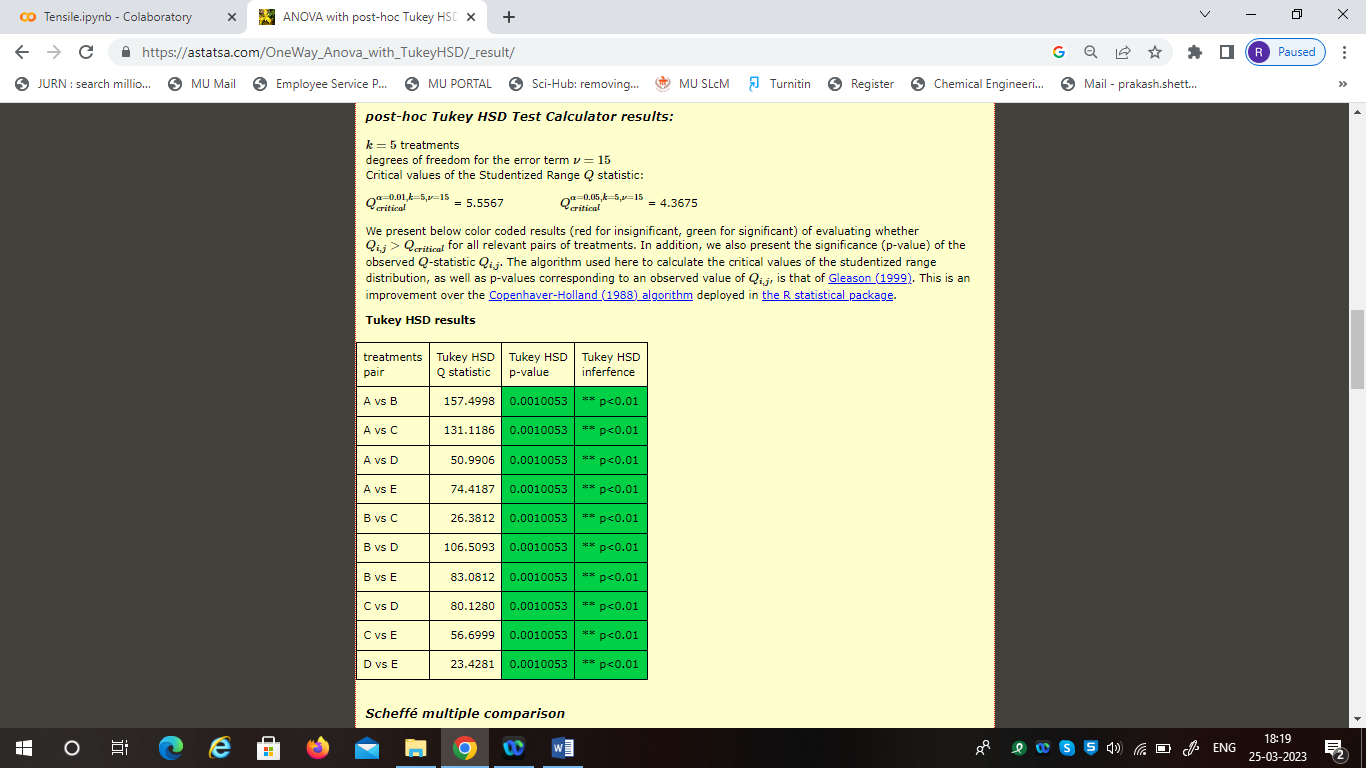


**Transparency**


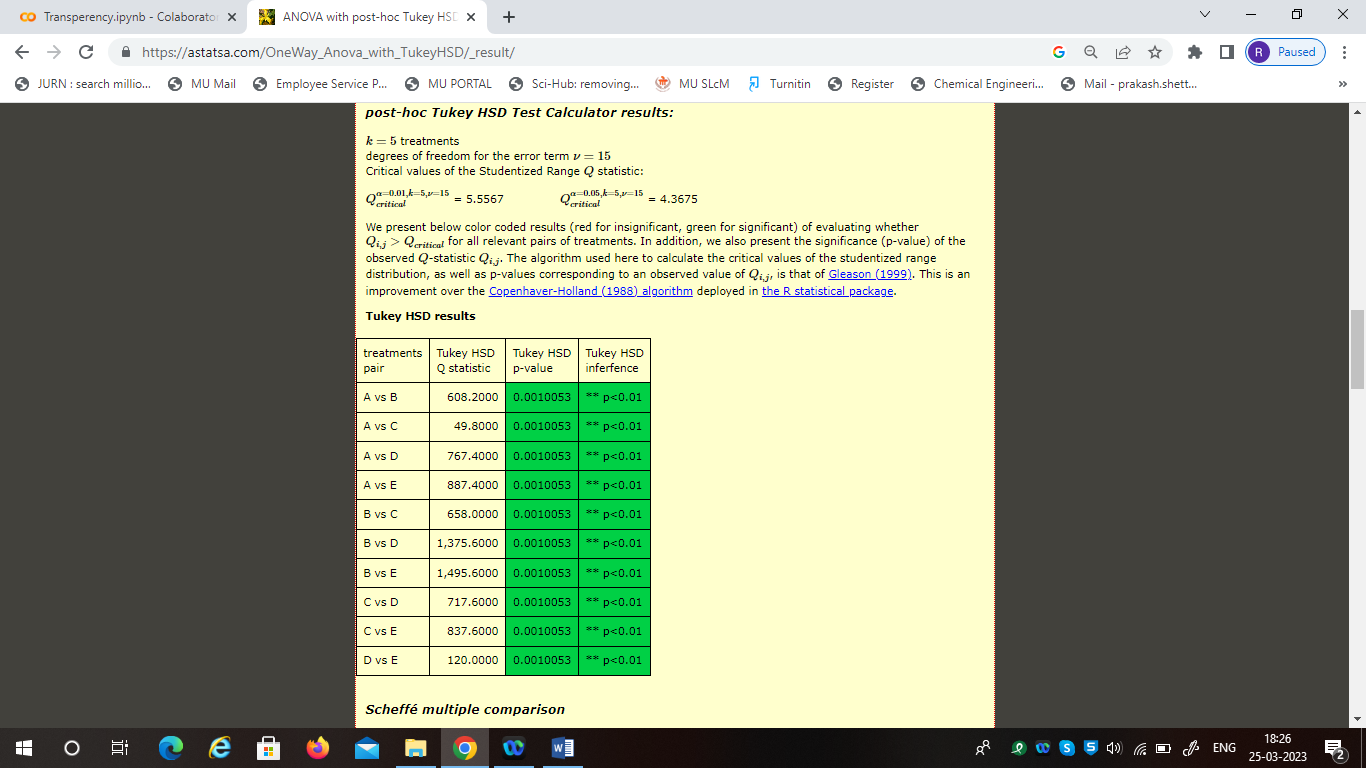


**Bursting strength**


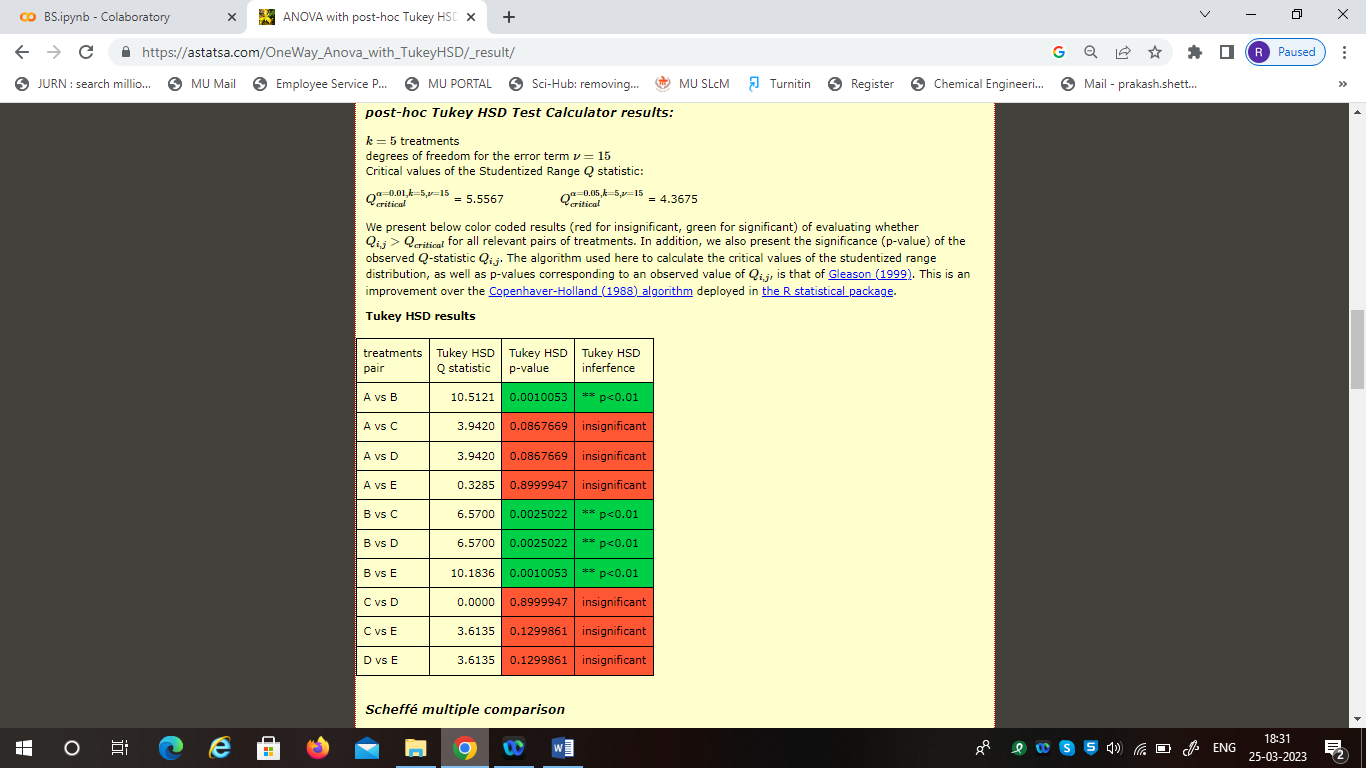


**Moisture content**


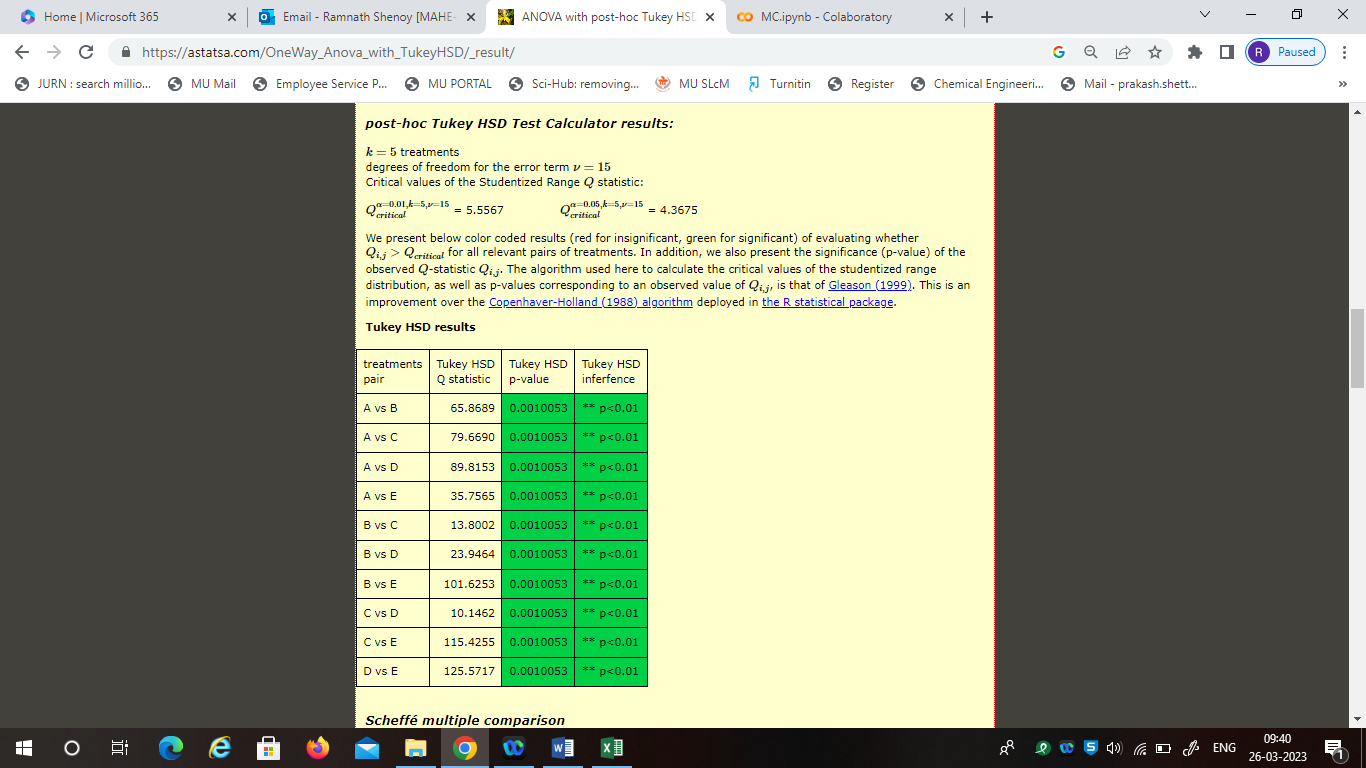


**Water solubility**


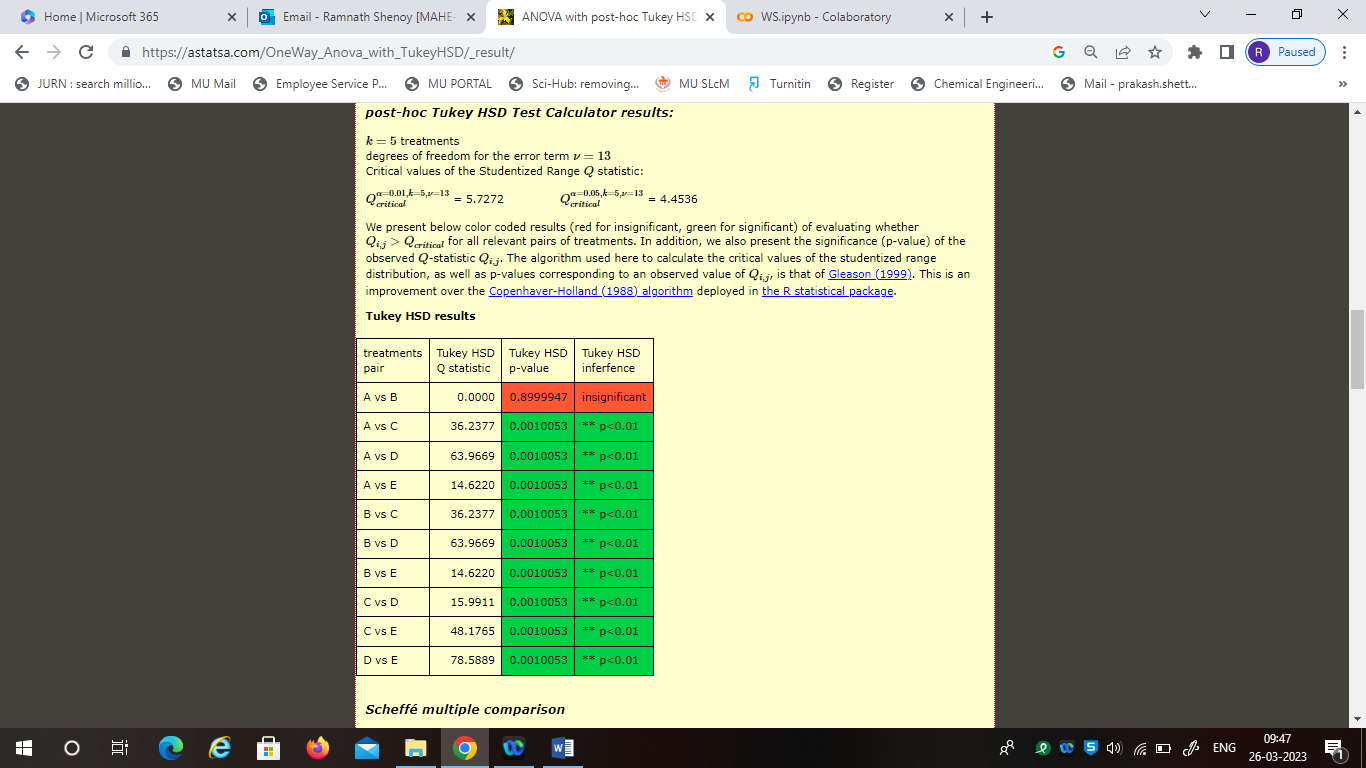


**Biodegradability**


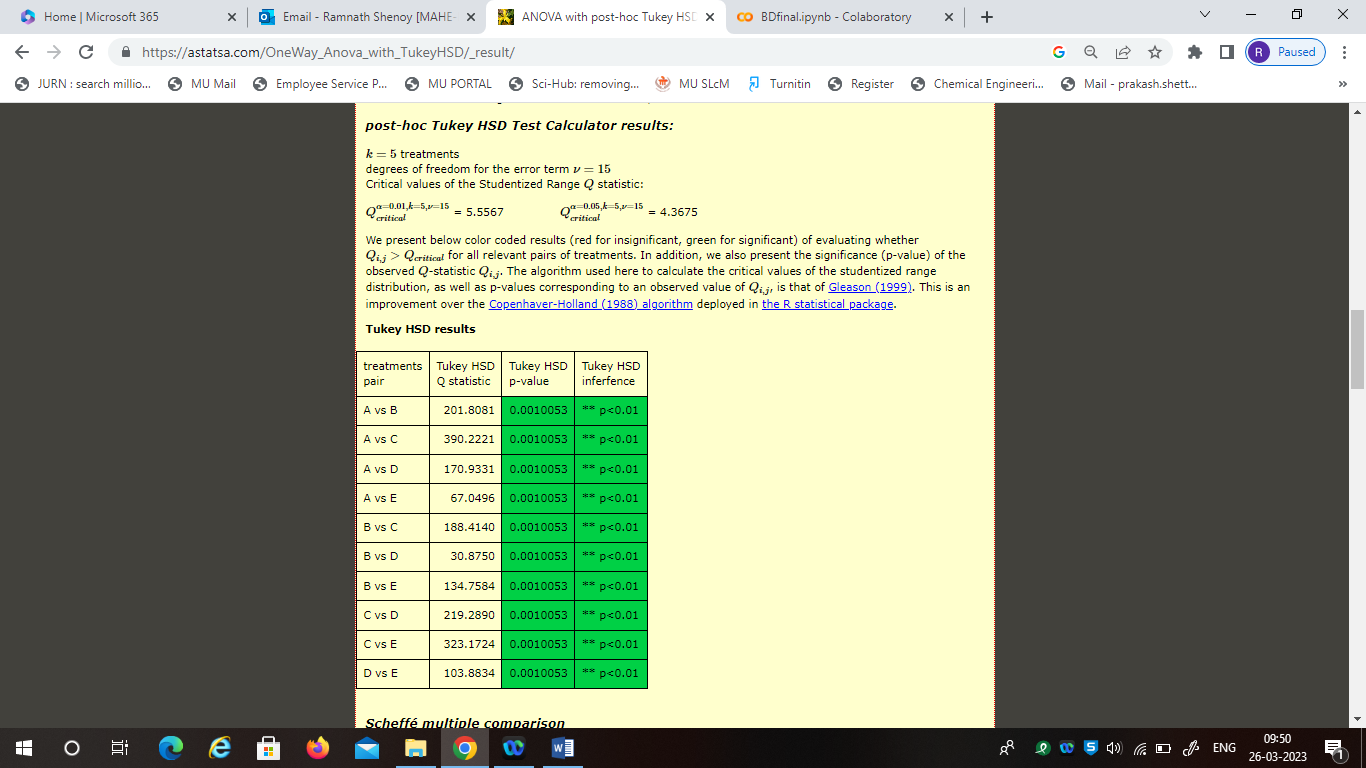

Supplement: Supplementary file 1 — Supplementary file1 (DOCX 2834 KB) [file 13197_2023_5803_MOESM1_ESM.docx]
